# Are there valid proxy measures of clinical behaviour? a systematic review

**DOI:** 10.1186/1748-5908-4-37

**Published:** 2009-07-03

**Authors:** Susan Hrisos, Martin P Eccles, Jill J Francis, Heather O Dickinson, Eileen FS Kaner, Fiona Beyer, Marie Johnston

**Affiliations:** 1 Institute of Health and Society, Newcastle University, 21 Claremont Place, Newcastle upon Tyne, NE2 4AA, UK; 2 Health Services Research Unit, University of Aberdeen, Health Sciences Building, Foresterhill, Aberdeen AB25 2ZD, UK; 3 Department of Psychology, University of Aberdeen, Health Sciences Building, Foresterhill, Aberdeen AB25 2ZD, UK

## Abstract

**Background:**

Accurate measures of health professionals' clinical practice are critically important to guide health policy decisions, as well as for professional self-evaluation and for research-based investigation of clinical practice and process of care. It is often not feasible or ethical to measure behaviour through direct observation, and rigorous behavioural measures are difficult and costly to use. The aim of this review was to identify the current evidence relating to the relationships between proxy measures and direct measures of clinical behaviour. In particular, the accuracy of medical record review, clinician self-reported and patient-reported behaviour was assessed relative to directly observed behaviour.

**Methods:**

We searched: PsycINFO; MEDLINE; EMBASE; CINAHL; Cochrane Central Register of Controlled Trials; science/social science citation index; Current contents (social & behavioural med/clinical med); ISI conference proceedings; and Index to Theses. Inclusion criteria: empirical, quantitative studies; and examining clinical behaviours. An independent, direct measure of behaviour (by standardised patient, other trained observer or by video/audio recording) was considered the 'gold standard' for comparison. Proxy measures of behaviour included: retrospective self-report; patient-report; or chart-review. All titles, abstracts, and full text articles retrieved by electronic searching were screened for inclusion and abstracted independently by two reviewers. Disagreements were resolved by discussion with a third reviewer where necessary.

**Results:**

Fifteen reports originating from 11 studies met the inclusion criteria. The method of direct measurement was by standardised patient in six reports, trained observer in three reports, and audio/video recording in six reports. Multiple proxy measures of behaviour were compared in five of 15 reports. Only four of 15 reports used appropriate statistical methods to compare measures. Some direct measures failed to meet our validity criteria. The accuracy of patient report and chart review as proxy measures varied considerably across a wide range of clinical actions. The evidence for clinician self-report was inconclusive.

**Conclusion:**

Valid measures of clinical behaviour are of fundamental importance to accurately identify gaps in care delivery, improve quality of care, and ultimately to improve patient care. However, the evidence base for three commonly used proxy measures of clinicians' behaviour is very limited. Further research is needed to better establish the methods of development, application, and analysis for a range of both direct and proxy measures of behaviour.

## Background

The measurement, reporting and improvement of the quality of health care provision are central to many current health care initiatives that aim to increase the delivery of optimal, evidence-based care to patients (*e.g.*, quality and outcomes framework (QOF) [1], new GMS contract [2]). In the UK, the new GMS contract [2] introduced in 2004 represents a growing trend towards pay-for-performance incentives in primary care, delivered through the QOF. Accurate measures of health professionals' clinical practice are therefore critically important not only to policy makers in guiding health policy decisions but also to practitioners in the evaluation of their own practice and to researchers both in identifying deficits and evaluating changes in the process of care.

Clinical practice can be measured directly – by actual observation of clinicians while practicing, or indirectly – by the use of a proxy measure, such as a review of medical records or interviewing the clinician. Direct measures include observation by a trained observer, video- or audio-recording of consultations, and the use of 'standardised' or 'simulated' patients. These are generally considered to provide an accurate reflection of the behaviour under observation, and as such represent a 'gold standard' measure of performance. However, direct measures are intrusive, can promote (unrepresentative) socially-desirable behaviour in the individuals being observed, and are time-consuming and costly to use, placing significant limitations on their use in any context other than small studies. Thus, they are not always a feasible option.

Measurement of clinical behaviour has therefore commonly relied on less costly and more readily available indirect sources of performance data, including review of medical records (chart review), clinician self-report, and patient report. Having effective and less costly proxy measures of behaviour could expand both the policy and research agendas to include important clinical behaviours that might otherwise go unexamined because of measurement difficulties. However, despite their widespread use, the extent to which these proxy measures of clinical behaviour accurately reflect a clinician's actual behaviour is unclear.

The aim of this review was to identify the current evidence relating to the relationships between direct measures and proxy measures of clinical behaviour. In order to establish whether any indirect measures can be used as proxies for actual clinical behaviour, the accuracy of medical record review, clinician self-reported and patient-reported behaviour were assessed relative to a direct measure of behaviour.

## Objective

The objective of the review was to assess whether there is a relationship between measures of actual clinical behaviour and proxy measures of the same behaviour, and how this relationship can best be described both on average and for individual clinicians.

## Methods

### Inclusion and exclusion criteria

We included any study that examined clinical behaviour (behaviour enacted by a clinician – doctor, nurses and allied health professionals – with respect to a patient or their care) within a clinical context. Studies were included if they reported a quantitative evaluation of the relationship between a direct measure representing actual behaviour and an indirect, proxy measure of the same behaviour. We excluded studies of undergraduate students. A direct measure of behaviour was defined as one based on direct observation of a clinician's actual behaviour in a clinical context by either a trained observer or a simulated patient, or of a video- or audio-recording of it. A proxy measure of behaviour was defined as one based on clinician self-report of recent or usual behaviour in a specified clinical situation, or patient-report of clinicians' behaviour or medical record review.

### Search strategy for identification of studies

The following databases were searched: PsycINFO (1840 to Aug 2004), MEDLINE (1966 to Aug wk 3 2004), EMBASE (1980 to Aug wk 34), CINAHL (1982 to Aug wk 3 2004), Cochrane central register of controlled trials (2004 issue 2), science/social science citation index (1970 to Aug 2004), current contents (social and behavioural med/clinical med) (1998 to Aug 2004), ISI conference proceedings (1990 to Aug 2004), and Index to Theses (1716 to Aug 2004). The search terms for behaviour, health professionals, and scenarios are shown in Table 1. The search strategy was devised to also identify studies for a related review that examined the relationship between intention and clinical behaviour, and hence contained the additional search term 'intention' [3]. The search domains were combined as follows: (Intention) AND (Behaviour) AND (health professionals), (Intention-behaviour) AND (health professionals), (behaviour) AND (outcomes) AND (health professionals). The reference lists of all included papers were checked manually.

**Table 1:** Keyword combinations for three domains, combined for the database search

**Behaviour**	**Health professionals**	Intention
Thesaurus headings: • BEHAVIOR • CHOICE BEHAVIOR • PLANNED BEHAVIOR • Behaviour?* • Clinician performance* • (Actor or abstainer) near behaviur*	(Intention or intend*) near behaviour?* Thesaurus headings: • HEALTH PERSONNEL • ATTITUDE OF HEALTH PERSONNEL • CLINICIANS Clinician* Counsellor* Dentist* Doctor* Family practition* General practition* GP*/FP* Gynaecologist* Haematologist* Health professional* Internist* Neurologist* Nurse* Obstetrician* Occupational therapist* Optometrist* OT* Paediatrician* Paramedic* Pharmacist* Physician* Physiotherapist* Primary care Psychiatrist* Psychologist* Radiologist* Social worker* Surgeon*/surgery Therapist*	Thesaurus heading: INTENTION • Intend* or intention* • Inclin* or disinclin*

Example thesaurus headings are given for the PsycINFO database and were adjusted and exploded as appropriate for other databases.

### Review methods

All titles and abstracts retrieved by electronic searching were downloaded to a reference management database; duplicates were removed, the remaining references were screened independently by two reviewers, and those studies which did not meet the inclusion criteria were excluded. Where it was not possible to exclude articles based on title and abstract, full text versions were obtained and their eligibility was assessed by two reviewers. Full text versions of all potentially relevant articles identified from the reference lists of included articles were obtained. The eligibility of each full text article was assessed independently by two reviewers. Disagreements were resolved by discussion or were adjudicated by a third reviewer.

### Quality assessment

#### External validity

External validity relates to the generalisability of study findings. We assessed this for included studies on the basis of:

1. whether the target population of clinicians was local, regional, or national.

2. whether the target population of clinicians was sampled or whether the entire population was approached – and if the population was sampled, whether it was a valid random (or systematic) sample – in order to assess the potential for selection bias.

3. the number of clinicians recruited and the total number of consultations assessed.

4. the percentage of participants enrolled for whom the relationship between direct and proxy measures of behaviour was analysed (attrition bias).

#### Internal validity

Internal validity relates to the rigor with which a study was conducted, and how confident we can be about any inferences that are subsequently made [4]. Important aspects of internal validity that are particularly relevant to the included studies are the reliability and validity of the measurement methods used to assess the performance of clinical behaviours. We therefore assessed internal validity on the basis of the psychometric evaluations performed by each study:

### Reliability

1. Measurement of inter-rater and intra-rater reliability for checklist scoring by trained observers and simulated patients.

2. Test re-test reliability of either direct or indirect measures.

### Validity of the scoring checklist

Content and face validity of the scoring checklist: *e.g.*, the rationale and process for the choice of items included and for any weights assigned to them;

### Validity of the direct measure method

General: The ability of the direct measure to accurately detect the aspects of behaviour under scrutiny (*e.g.*, the range of clinical actions on the scoring checklist).

### Simulated patients

1. Content validity of simulated cases: the level of correspondence between components of simulated cases and actual clinical presentations of the condition in question.

2. Face validity: judgments made by individuals other than the research team that the simulated case 'looks like' a valid case representation of the clinical condition in question.

3. Training of simulated patients in the case protocol.

4. Assessment of cueing and reporting of detection of simulation.

### Validity of the Proxy methods

#### Patient vignettes

Content validity: Correspondence between the operationalisation of the simulated case in the standardized patient protocols and written vignettes.

#### Patient report and Clinician self-report

Content validity: Correspondence between the content and wording of items on the scoring checklist and the items on the questionnaire or interview schedule.

### Appropriateness of the statistical methods used

The studies included in the current review used a range of statistical methods to summarise and compare direct and proxy measures of behaviour. To help us synthesise the data from included studies we conducted a companion review to assess the appropriateness of the different statistical methods they used (Dickinson HO et al. Are there valid proxy measures of clinical behaviour? Statistical considerations, submitted). Our conclusions are summarized below.

The included studies were based on recording whether a clinician performed one or more clinical actions that we refer to as 'items'. Some studies compared direct and proxy measures 'item-by-item'; other studies combined items into summary scores and then compared direct and proxy summary scores.

Statistical methods used by studies that compared direct and proxy measures item-by-item included: sensitivity and specificity; total agreement; total disagreement; and kappa coefficients. For these studies, we concluded that sensitivity and specificity were generally the best statistics to assess the performance of a proxy measure, provided these statistics were not based on a combination of items describing different clinical actions.

Statistical methods used by studies that compared summary scores included: comparisons of means; analysis of variance (ANOVA); t-tests; and Pearson correlation. For these studies, we concluded that summary measures should capture a single underlying aspect of behaviour and measure that construct using a valid measurement scale. The average relationship between the direct and proxy measures should be evaluated over the entire range of the direct measure, and the variability about this average relationship should also be reported. Hence, comparisons of mean scores are inappropriate. ANOVA and t-tests are likewise inappropriate because they are essentially methods of testing whether the mean score is the same in both groups. Correlation is inappropriate because it cannot assess whether there is systematic bias in the proxy measure (*i.e.*, whether the proxy measure consistently under- or overestimates performance by a certain amount). Furthermore, the strength of the estimated correlation depends on the range of scores of the proxy and direct measures.

### Data extraction

For each study, we extracted the: age and professional role of participants; behaviour assessed; quantitative data measuring the relationship between the direct and proxy measures of behaviour; method of measuring behaviour and psychometric properties of measure; and quality criteria specified above.

### Evidence synthesis

For studies that reported single binary (yes/no) items, we extracted, if possible, the number of consultations for which: both the direct and proxy measures recorded the item as performed (true positives); both the direct and the proxy measures recorded the item as not performed (true negatives); the direct measure recorded the item as performed but the proxy measure did not (false negatives); and the direct measure recorded the item as not performed but the proxy measure recorded it as performed (false positives).

We estimated the mean and 95% confidence intervals (CI) for the sensitivity, specificity, and positive predictive value of the item and present these on forest plots. If studies did not report the above numbers but reported the sensitivity and/or specificity, these statistics were extracted. For all studies for which their mean values were available, the sensitivity was plotted against the false positive rate (1-specificity) because studies which fall in the top left of this plot are generally regarded as having better diagnostic accuracy (high sensitivity and high specificity); however, a summary ROC curve was not fitted to plots due to the heterogeneity between studies in behaviour measured and methods of measurement. Where possible, we also calculated the positive and negative predictive values for individual items.

For studies that reported aggregated scores summarising several items, we extracted any statistics presented that summarised the mean and variance of the direct measure and/or proxy summary scores and the relationship between the direct measure and proxy.

## Results

### Description of included studies

**Figure 1:**
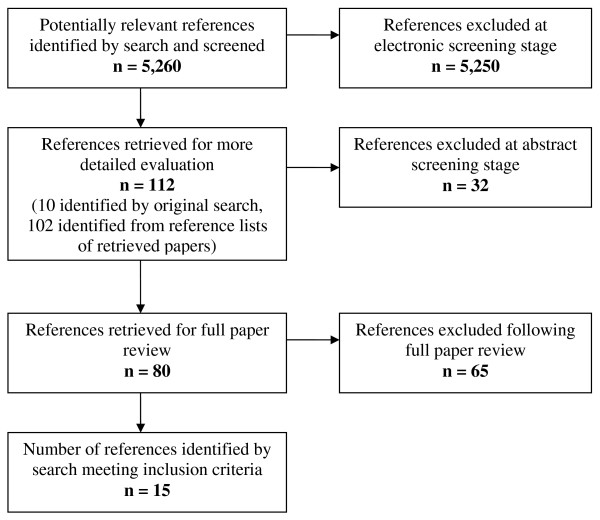
**Identification of included references (QUORUM diagram)**.

The search strategy identified 5,260 references (Figure 1). The titles and abstracts of these references were screened independently by two reviewers. Ten papers were retrieved for full text review and their reference lists screened for other potential papers. A further 102 papers were identified from the reference lists of retrieved papers, their abstracts were again reviewed independently by two reviewers, and 41 of these were retrieved for full text review. Fifteen papers, based on comparisons from eleven separate source studies, fulfilled the inclusion criteria and their data were abstracted [5-19]. As papers reporting different findings from the same study [5,6,10,12,14,18] present different data and, with the exception of two [10,18], used different methods of analysis, we have considered them as 15 separate reports for the purpose of this review.

For the 15 reports, 771 clinicians were enrolled and proxy measures of the clinical behaviour of 717 (93%) clinicians were evaluated relative to a direct measure. A summary of the characteristics of the 15 included reports is presented in Table 2, with further detail presented in Additional File 1. Ten reports originated in the United States, two in the Netherlands and one each in the United Kingdom, Australia, and Canada. The aim of 12 of 15 reports was to validate or to assess the 'accuracy' of an indirect measure of clinician behaviour relative to a specific direct measure. The aim of the remaining three reports was to assess the relative validity of different measures (both indirect and direct) to each other.

**Table 2:** Summary of included study characteristics and clinical behaviours measured

**Study**	**Characteristics**							**Behaviour measured**		
	**1. Type of participants** **2. Target population** **3. Sampling strategy**	**Participants approached & analysed**			**Consultations/sessions/indications observed/vignettes completed & analysed**			**1. Clinical area/s** **2. Behaviour/s observed** **(No. of clinical actions scored)**	**No. of checklist** **items**	**Summarised** **(weighted)**
		**N**	**n**	**%**	**N**	**n**	**%**			

**Stange **[5] **1998**	1. Family practice physicians 2. Members of the Ohio Academy of FPs, practice within 50 miles radius of Cleveland & Youngstown 3. Convenience sample	138	128	93	4454	4432 (MR) 3283 (PR)	99 (MR) 74 (PR)	1. Delivery of a range of outpatient medical services 2. Counselling (29), physical examination (16), screening (5), Lab tests (10), immunisation (7), Referral (4)	79	
**Flocke **[6] **2004**	1. Family physicians 2. Primary care physicians in North West Ohio 3. All physicians approached	138	128	93	4454	2,670	60	1. Health promotion 2. Smoking (2), alcohol, exercise, diet, substance use, sun exposure, seatbelt use, HIV & STD prevention	10	
**Wilson **[7] **1994**	1. General practitioners (GPs) 2. 10 general practices in Nottinghamshire 3. Selection of GPs not reported. Minimum of two non-random consultations were recorded	16	16	100	3324	516 (MR) 335 (PR)	16 (MR) 10 (PR)	1. Health promotion 2. Asked patient about 4 health behaviours: smoking (1), alcohol (1), diet & exercise (1); measurement of blood pressure (1)	4	
**Ward **[8] **1996**	1. Post-graduate trainees 2. Training general practices in New South Wales 3. Trainees who were having their first experience in supervised general practice	34	34	100	1500	1075	72	1. Smoking cessation 2. Establish smoking status & provide smoking cessation counselling (2)	2	
**Zuckerman **[9] **1975**	1. Paediatricians 2. Physicians working in a university medical centre serving an inner-city population 3. All 3 staff physicians	3	3	100	51	51	100	1. Paediatric consultation 2. Diagnosis and management (8), historical items (7)	15	
**Luck **[10] **2000**	1. Primary care physicians 2. 2 general internal medicine primary care outpatient clinics 3. Random sample of 10 physicians at each site	20	20	100	160	160	100	1. Management of LBP, DM, COPD, CAD. 2. History, Physical exam, Tests ordered, Diagnosis & Treatment/management (21 for LBP)	NR	√ (w)
**Page **[11] **1980**	1. Community pharmacists 2. Participants on a continuing education course in British Columbia, Canada 3. All participants	30	30	100	58	58	100	1. Management of: Cold, Pain 2. Recommend either: non-prescription medication (cold = 17, pain = 15) or see physician (cold = 17, pain = 18)	103	√ (w)
**Gerbert **[12] **1988**	1. Primary care physicians 2. Primary care physicians serving 6 counties in California 3. Convenience sample	63	63	100	197	197	100	1. Medication regimens in the management of COPD 2. Prescription of theophyllines (1), sympathomimetics (2), oral corticosteroids (1)	4	
**Pbert **[13] **1999**	1. Primary care physicians 2. Attending physicians & their patients at University medical centre in Massachusetts. 3. Convenience sample	12	12	100	154	108	70	1. Smoking cessation 2. Cessation counselling (15)	15	√
**Gerbert **[14] **1986**	1. Primary care physicians 2. NR 3. Convenience sample	63	63	100	214	192	90	1. Management of COPD 2. Symptoms (8), signs (2), Tests (3), Treatments (3), Patient education (4)	75	√
**Dresselhaus **[15] **2000**	1. Primary care physicians 2. 2 general internal medicine primary care outpatient clinics 3. Random sample of 10 physicians at each site	20	20	100	160	160	100	1. Management of low back pain, diabetes mellitus, COPD, CAD. 2. Preventive care: tobacco screening (1), smoking cessation advice (1), prevention measures (1), alcohol screening (1), diet evaluation (1), exercise assessment (1) & exercise advice (1)	7	√
**Rethans **[16] **1987**	1. GPs 2. GPs working in Maastricht 3. All participants	55	25	46	27	25	93	1. Management of Urinary Tract Infection 2. History taking (8); Physical Examination (3); Instructions to patients (7); Treatment (2); Follow-up (4)	24	√
**Rethans **[17] **1994**	1. GPs 2. Sampling strategy reported elsewhere. 3. Sampling strategy reported elsewhere	39	35	90	140	101	72	1. Management of tension headache; acute diarrhoea; pain in the shoulder; check-up for non-insulin dependent diabetes. 2. History, Physical exam, Lab exam, Advice, Medication & follow-up (range over 4 conditions: 25–36)	25–36	√
**Peabody **[18] **2000**	1. Primary care physicians 2. 2 general internal medicine primary care outpatient clinics 3. Random sample of 10 physicians at each site	20	20	100	160	160	100	1. Management of low back pain (LBP), diabetes mellitus (DM), Chronic obstructive pulmonary disease (COPD) oronary artery disease (CAD). 2. History taking (7), Physical examination (3), lab tests (5), Diagnosis(2), Management (6) (Averaged 21 actions per case)	168	√ (w)
**O'Boyle **[19] **2001**	1. Nurses 2. ICU staff in 4 metropolitan teaching hospitals in "Mid-West" USA 3. ICUs with comparable patient populations	124	120	97	120	120	100	1. Adherence to hand hygiene recommendations 2. Hand washing (for a maximum of 10 indications)	1	√

Participants in 12 reports were primary care physicians [5-8,10,12-18]; in other reports participants were nurses [19], community pharmacists [11], and paediatricians [9].

### Clinical behaviours

Five reports considered a range of clinical behaviours (*e.g.*, history taking, physical examination, ordering of laboratory tests, referral, diagnosis, treatment, patient education, and follow-up) in relation to the management of a variety of common out-patient conditions: urinary tract infection (UTI) [16]; tension headache, acute diarrhoea, and pain in the shoulder [17]; coronary artery disease (CAD), low back pain, and chronic obstructive pulmonary disease (COPD) [10,14,18]; diabetes [10,17,18]. One report considered the behaviour of recommending non-prescription medication or physician visit for common cold and pain symptoms [11], and one report evaluated medication regimens prescribed for patients with COPD [12]. Six reports considered health promotion behaviours, *e.g.*, giving advice about: smoking cessation [5-8,13,15]; alcohol use, exercise, and diet [5-7]; preventive care in relation to CAD, low back pain, and COPD [15]; and sun exposure, substance use, seatbelt use, and sexual health [6]. One report considered the provision of a wide range of outpatient services including counselling, screening, and physical examination [5]; and one evaluated physician communication in paediatric consultations [9]. One report considered hand hygiene [19].

With the exception of two studies [8,13], the clinical behaviours measured were 'necessary' or 'recommended' clinical actions categorized as such according to either national guidelines or expert consensus. Four studies also included actions that were unnecessary or that should not be performed (*e.g.*, prescribing an antibiotic for a viral infection) [10,11,16,18].

### Methods used for measuring clinical behaviour

In all studies a checklist was used to record the performance of clinical actions relevant to the clinical area studied. All clinical actions were discrete activities, that is, could be coded as 'yes' or 'no' (*e.g.*, the recording of blood pressure, asking about smoking habits). The number of possible clinical actions observed in each study ranged from one [19] to 168 [18].

A summary of the proxy and direct measures used by the 15 included reports is presented in Table 3, with further detail presented in Additional File 2. The direct measure of clinical behaviour was based on either: post-encounter reports from simulated patients, [10,11,15-18]; prospective reports made by trained observers during direct observation of actual consultations[5,6,19]; or post-encounter reports from trained observers rating audio- or video-recordings of consultations [7-9,12-14].

**Table 3:** Summary of the measures used by included studies, methods of analysis and results of comparisons

**Study**	**Proxy measure**				**Direct Measure (DM)**			**Analysis**			
	**Description** **1. Method** V = Clinical vignette (No. of case simulations) CI/Q = Clinician interview/questionnaire MR = Medical Record review PI/Q = Patient interview/questionnaire **2. Timing**	**Clinician self report (SR)**	**Medical Record Review (MR)**	**Patient report (PR)**	**Description** **1. Method** SP = Simulated Patients DO = Direct Observation VR = Video recording AR = Audio recording **2. Timing**	**SP Training reported**	**Psychometrics (IRR)**	**Compared Item by Item**	**Compared Summary Scores**	**Agreement between measures:** Co-efficient r; kappa (k); Structural equation modelling (SEM); Sensitivity (Sens) & Specificity (Spec) **Difference between mean scores:** ANOVA; T-test	**P**

**Stange **[5] **1998**	1. MR; PQ 2. At end of consultation		√	√	DO		0.39 to 1.00 (kappa)	√		MR Sens = 8% (diet advice) – 92% (Lab tests) Spec = 83% (social history) – 100% (counselling services, physical exam, lab tests) k = 0.12 to 0.92 (79 comparisons) PR Sens = 17% (mammogram) – 89% (Pap test) Spec = 85% (in-office referral) – 99% (immunisation, physical exam, lab tests) k = 0.03 to 0.86 (53 comparisons)	NR
**Flocke **[6] **2004**	1. PQ 2. At end of consultation (24%) or postal return (76%)			√	DO		NR	√		Sens* = 11% (substance use) – 76% (smoking cessation)	NA
**Wilson **[7] **1994**	1. MR; PQ 2. At end of consultation		√	√	AR		0.79 to 1.00	√		MR Sens = 31%, Spec* = 99% 28.6 (Alcohol) Sens = 29%, Spec* = 100% 83.3 (BP) Sens = 83%, Spec* = 93% % agreement between DM & MR: 45.5 (Smoking) PR Sens = 74%, Spec* = 94% 75.0 (Alcohol) Sens = 75%, Spec* = 94% 100 (BP) Sens = 100%, Spec* = 90% % agreement between DM & PR: 81.8 (Smoking)	NA
**Ward **[8] **1996**	1. PQ 2. Questionnaire mailed to patient within 2 days of consultation			√	AR		0.74 to 0.94 (kappa)	√		Sens = 93% (smoking status) Spec = 79% Sens = 92% (cessation advice) Spec = 82%	NA
**Zuckerman **[9] **1975**	1. MR 2. At end of consultation		√		AR		NR	√		Sens* = 0% (side effects) – 100% (Diagnosis) Spec* = 9% (Diagnosis) – 100% (side effects)	NA
**Luck **[10] **2000**	1. MR 2. At end of consultation		√		SP (27) each role-playing 1 of 8 case simulations	√	NR	√	√	ANOVA (4-way) Necessary care: Sens = 70%, Spec = 81% Unnecessary care: Sens = 65%' Spec = 64%.	<0.0001 NA
**Page **[11] **1980**	1. V (4) 2. Upto 6 weeks before or 3 weeks after SP visit	√			SP (4) each role-playing 1 case simulation	√	0.76	√	√	r = .56 & .68 r = .26 & .37 "Must do" actions Sens* = 97%, Spec* = 33% "Must not do" actions Sens* = 30%, Spec* = 98%	>0.05 <0.05
**Gerbert **[12] **1988**	1. CI; MR; PI 2. At end of consultation	√	√	√	√R		NR	√		k = 0.67 (SR) k = 0.54 (MR) k = 0.50 (PR)	<0.001 <0.001 <0.001
**Pbert **[13] **1999**	1. CI; PI 2. At end of consultation	√		√	AR.		NR	√	√	r = 0.77 (SR) r = 0.67 (PR)	<0.0001 <0.0001
**Gerbert **[14] **1986**	1. CI; MR; PI 2. At end of consultation	√	√	√	√R		0.52 to 0.93 (kappa)	√		Median % agreement (All categories): 0.84 (SR) 0.88 (MR) 0.86 (PR)	NA
**Dresselhaus **[15] **2000**	1.V (8); MR 2. NR	√	√		SP (4) each role-playing a simple and complex case presentation	√	NA	√		ANOVA (3-way)	<0.01
**Rethans **[16] **1987**	1. V (1). 2. Completed 2 months after SP visit	√			SP (3) each role-playing same case simulation	√	0.78 to 1.0 (kappa)	√	√	T-test: Overall "Obligatory" "Intermediate" "Superfluous"	ns <0.005 <0.05 <0.05
**Rethans **[17] **1994**	1. MR 2. Charts reviewed two years after SP visit.		√		SP (4) each role-playing 1 of 4 case simulations	√	0.93 (kappa)	√	√	r = 0.54 (Overall) r = 0.17 (History taking) r = 0.45 (Physical exam) r = 0.75 (Lab exam) r = 0.50 (Advice) r = 0.43 (Medication) r = -0.04 (Follow-up)	<0.05) ns ns <0.01 <0.05 ns ns
**Peabody **[18] **2000**	1. V (8); MR 2. Completed "several weeks" after SP visit	√	√		SP (4) each role-playing a simple and complex case presentation	√	NA		√	ANOVA (4-way)	<0.001
**O'Boyle **[19] **2001**	1. % time practiced hand hygiene 2. Up to one month prior to observation period	√			DO Nurses observed for 2 hours or until 10 indications for handwashing had occurred		0.94 to 0.98		√	r = 0.21 SEM = 0.201	<0.05 <0.05

* Calculated by authors NA = Not applicable NR = Not reported ns = non-significant

The proxy measure of clinical behaviour was based on either: clinician self-report of recent behaviour on self-completion questionnaire or by exit interview [5,12-14,19]; clinician self-report of simulated behaviour in a specified clinical situation using clinical vignettes [11,15,16,18]; medical record review [5,7,9,10,12,14,15,17]; patient report on self-completion questionnaire or by exit interview [5-8,12-14]; or eight reports evaluated multiple proxy measures [5,7,9,12-15,19].

### Methodological quality of included studies

#### External validity

The target populations in nine reports were regional [5,6,8,11,12,14,16,17,19]; all other reports targeted local populations, such as physicians in two general internal primary care outpatients clinics [10,15,18], attending physicians at a university medical centre [9,13], and general practitioners in ten general practices [7]. Six reports approached all participants in their target population [6,7,9,11,16,17], three randomly sampled a group of clinicians [10,15,18], and six used convenience sampling [5,8,12-14,19]. The number of clinicians enrolled and analysed in each report ranged from three [9] to 138 [5,6] (median 34). Ten reports retained and analysed 100% of recruited clinicians [7-15,18]. The median number of consultations observed was 160, with a range from 27 [16] to 4,454 [5,6]. For further details see Additional File 2.

#### Internal validity

##### Validity of the checklists used

In six reports, the content of the checklist was based on national guidelines for the behaviour in question [5,6,10,15,18,19], and for a further six reports content was derived by expert consensus [11-14,16,17]. Two reports asked simply whether or not a physician asked about a particular lifestyle behaviour (*e.g.*, smoking), and whether or not they offered counselling [7,8]. One report did not report the rationale for their choice of clinical actions [9]. Inter-rater reliability for assignment of weights to individual checklist items was presented in one report [11] and was 0.73.

An important criterion for validity is that a measure should be reliable. Inter-rater reliability of scores generated from checklists using direct measures were reported for eight of the 15 included reports [5,7,8,11,14,16,17,19], and ranged from 0.39 [5] to 1.00 [5,16] (Table 2). Five additional reports evaluated the reliability of scoring between raters – stating these to be 'good' – but did not present inter-rater reliability statistics [6,10,13,15,18]. Two reports presented intra-rater reliabilities which were 0.78 to 0.96 [16] and 0.74 to 1.0 [8]. Two reports did not discuss the reliability of the scoring procedure [9,12]. One report evaluated the reliability of the proxy measures used [16].

##### Validity of the direct methods used

Only one report presented assessment of the ability of the direct measure to detect the behaviours of interest [14]. They found that videorecording captured a median of 48% of the content of the overall consultation observed, but that the level of capture varied from 10% to 100% depending on the clinical action.

Of the six reports that used standardised patients as the direct measure, four assessed the content and face validity of the patient scripts using expert review [10,15,18]. All reported that training was provided to standardised patients, but two reports did not provide detail about the duration or nature of the training [16,17]. In three studies, standardised patients were experienced actors, who were trained according to a published protocol which was delivered by experienced university-based educators [10,15,18]. One report used graduate students who were trained for four hours as standardised patients [11]. The experience of the trainer was not reported, but standardised patients pilot tested one of their simulated roles with a community pharmacist, and their checklist ratings were compared across four videotaped standardised patient encounters with pharmacists. Three reports reported detection rates of the standardised patient (*i.e.*, the clinician realised that standardised patients were not genuine patients), and these were low (3%) [10,15,18].

##### Validity of the proxy methods used

With the exception of one report [19], the proxy method was directly related to the study visit; for example, reports using medical record review as the proxy method abstracted medical records pertaining only to the study visit, or patients were asked about a specific consultation. The proxy measure used by O'Boyle *et al. *[19] was collected two weeks to four months before the direct measurement.

In four reports that compared performance on the direct measure with a written vignette [11,15,16,18], all but one [11] reported these to be identical case matches. In the latter report, two standardised patient case protocols differed from the corresponding written vignette in the nature of the clinical complication presented by the standardised patient [11]. The correspondence of standardised patient and vignette case protocols for two reports was not reported [10,17].

### Appropriateness of statistical methods used to summarise and report the relationship between direct and proxy measures

#### Studies comparing items

Thirteen reports compared measures of behaviour item-by-item [5-17]. Four of these studies estimated the sensitivity of the proxy measure for each clinical action measured [5-8], two the specificity [5,8] and one [7] the false positive rate from which we calculated specificity. It was possible to calculate the sensitivity and specificity for individual clinical actions from the raw data presented in a further report [9]. Three studies grouped clinical actions into categories: 'necessary' and 'unnecessary' actions [10]; 'must do', 'should do', 'must not do' and 'should not do' actions [11]; and 'essential' and 'intermediate' actions [17]. Luck *et al. *[10] then estimated the sensitivity and specificity within each category, and it was possible to estimate the sensitivity and specificity for each category specified by Page *et al. *[11] from the raw data presented. Rethans *et al. *[17] also calculated the sensitivity of each item (referred to by the authors as 'content scores') but reported only the mean and inter-quartile range of sensitivities within each clinical area. Hence, sensitivities were available for seven studies and specificities for six studies.

Six reports comparing item-by-item used other statistical methods to compare their data [12-17]. These studies assessed 'agreement' and/or 'disagreement' between measures; five reported agreement as the percentage of recommended behaviours performed as recorded on the direct and proxy measures [7,12,13,15,16], one also reported disagreement as the proportion of behaviours not recorded by the proxy measure that were detected by the direct measure [12]; and one study estimated the 'total agreement' and 'total disagreement' between measures, reporting median 'convergent validity' for 20 individual items and five clinical categories [14].

#### Studies comparing summary scores

Seven reports aggregated items into summary scores of clinicians' behaviour [10,11,13,16-19]. Three studies used ANOVA to compare summary scores [10,13,18]; one study used paired t-tests [16]; and four studies reported Pearson correlation coefficients [11,13,17,19].

### Relationship between direct and proxy measures behaviour

#### Studies comparing items

##### Patient report

Three reports comparing item-by-item and reporting sensitivity and specificity [5,7,8], and one reporting sensitivity only [6], examined patient report as a proxy measure of clinician performance. Measurement techniques used were either patient questionnaire or patient interview, which were compared with direct observation [5,6] and audio-recording [7,8] (Table 2).

**Figure 2:**
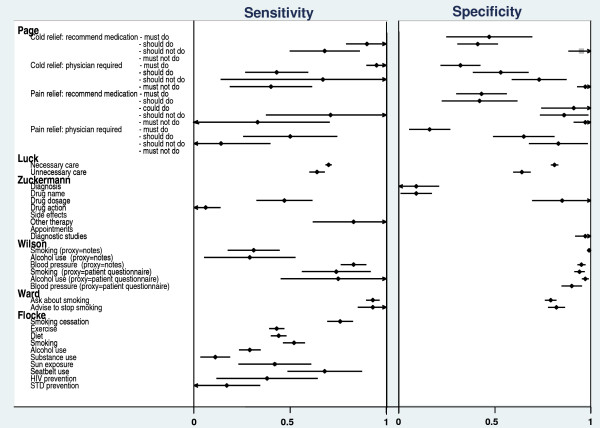
**Sensitivities and specificities for six studies**.

Median sensitivities for clinical actions relating to the provision of general outpatient services [5] and for health advice on a range of patient behaviours [6] were 53% (range 25 to 89) and 43% (range 11 to 76), respectively. Sensitivities for: the provision of smoking cessation advice were 74% [7], 93% [8], and 76% [6]; for asking about alcohol use they were 75% [7] and 29% [6], and 100% for measuring blood pressure [7] (Figure 2). Median specificity for patient report was 98% (range 83% to 99%) [5,7,8] across a number of services, 79% [8] and 94% [7] for smoking cessation counselling, and 90% for the measurement of blood pressure [7] (Figure 2).

**Figure 3:**
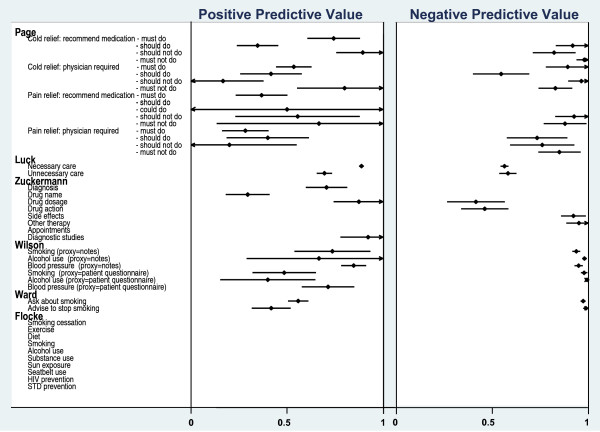
**Positive and Negative Predictive Values for six studies**.

Positive and negative predictive values could be calculated from the raw data of two reports evaluating the provision of smoking and alcohol advice and the measurement of blood pressure [7,8]. The positive predictive values for patient-report were: 0.49 [7], 0.42, and 0.55 [8] for smoking advice; 0.40 for alcohol advice [8]; and 0.70 for the measurement of blood pressure [7,8] (Figure 3). The negative predictive values for patient-report of the same behaviours were high for both studies (>0.90) [7,8]. This would suggest that patients accurately reported not receiving advice and not having their blood pressure measured, but they are less accurate in reporting that clinicians did perform these behaviours.

Three further reports compared item-by-item but did not report sensitivity or specificity for their data [12-14]. Gerbert *et al. *[14] report a median 'total agreement' of 86% between measures for the performance of clinical actions relating to the management of COPD. Gerbert *et al. *[12] present a kappa coefficient of 0.50 for the level of concordance between patient report and their direct measure of video-recording and a 'disagreement' between the measures of 24%. Pbert *et al. *[13] made comparisons across measures for the detection of individual items using Cochrane's *Q *tests. These comparisons suggested that patients tended to over-report their clinician's behaviour compared to the direct measure of audio-recording.

##### The accuracy of patient-report

**Figure 4:**
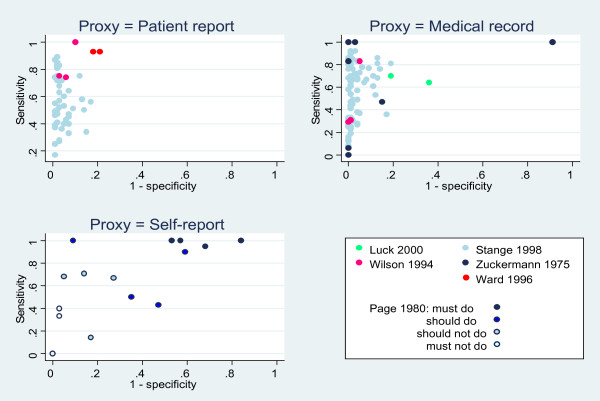
**ROC plots of sensitivities and specificities for three proxy measures**. Behaviours/actions in the top left-hand quadrant have both high sensitivity and specificity. See Stange 1998 [5] for additional sensitivities and specificities for 78 items.

ROC curves were plotted for the three studies where both sensitivity and specificity were available [5,7,8](Figure Figure 4). The accuracy of patient report varied according to the clinical action of interest. Performance of the behaviours located in the top-left quadrant of this plot were reported most accurately by patients. These included the provision of counselling for health behaviours such as smoking, alcohol use, seat belt use, and breast self-examination, which were more accurately reported by patients than the provision of counselling for accident prevention, dental health, contraception, and exercise (behaviours located in the bottom-left quadrant). The accuracy of patient report for clinical actions relating to physical examination, laboratory tests, and screening services also varied with the type of examination, test, or service undertaken [5].

### Medical record review

Four reports comparing item-by-item and reporting sensitivity and specificity compared medical record review with direct observation in one report [5], with audio-recording in two reports [7,9], and standardised patient accounts in one report [10], (Table 2).

Median sensitivity for a range of clinical actions relating to the provision of general outpatient services was 60% (range 8% to 92) [5] and 83% (range 0 to 100%) [9] for clinical actions undertaken during routine patient consultations (Figure 2). For smoking cessation advice, alcohol counselling and the measurement of blood pressure sensitivities were 31%, 29%, and 83%, respectively [7], and for 'necessary' and 'unnecessary' actions sensitivities were 70% and 65%, respectively [10] (Figure 2). Median specificity for medical record review across a number of services was 90% (range 81% to 100%) [5], and 97% (range 9% to 100%) [9]. Specificities for smoking counselling, alcohol counselling, and the measurement of blood pressure were 99%, 100%, and 93%, respectively [7], and 64% and 81% for 'necessary' and 'unnecessary' actions, respectively [10] (Figure 2).

As the raw data were available for three reports evaluating medical record review [7,9,10], it was possible to calculate a range of positive and negative predictive values for this proxy method (Figure 3). The positive predictive ability of medical record review ranged from 0.30 to 0.92 (Median = 0.86) across different clinical actions, and was highest for 'necessary' care items (PPV = 0.85) [10], recording of drug dosage (PPV = 0.88), diagnostic behaviours (PPV = 0.91) [9], and the measurement of blood pressure (PPV = 0.84) [7] (Figure 3). The negative predictive ability of medical record review ranged from 0.39 to 1.00 (Median = 0.73) across different clinical actions, and was lowest (<0.50) for the recording of drug dosages and drug action [9], and highest for advice-giving behaviours and the measurement of blood pressure [7] (Figure 3).

Four further reports compared item-by-item but used other statistical methods to do this [12,14,15,17]. Gerbert *et al. *(1986) [14] report total agreement of 88% between medical record review and video-recording for behaviours relating to the general management of COPD. Gerbert *et al. *(1988) [12] present a kappa coefficient of 0.54 for the level of concordance between medical record review and video-recording, and a total disagreement between these measures of 21%. Rethans *et al. *[17] and Dresselhaus *et al. *[15] presented summary percentage scores (65.6%, 54.0%, and 45.8%, respectively) that were consistently lower than scores reported by a standardised patient (76.2%, 68.0%, and 61.7%, respectively). Rethans *et al. *[17] also reported a correlation coefficient of r = 0.54 between summary scores relating to the management of commonly presenting outpatient conditions (Table 2).

### The accuracy of medical record review

ROC curves were plotted for four studies where both sensitivity and specificity were reported [5] or could be calculated from the raw data presented [7,9,10] (Figure 4). The accuracy of medical record review varied according to the type of clinical behaviour or action that was being measured. Review of medical records yielded more accurate estimates of clinician performance for actions relating to physical examination, blood pressure measurements, laboratory tests, and screening services (which were located in the top-left quadrant) than for actions relating to the provision of a wide range of counselling services, including smoking cessation advice, and alcohol counselling.

### Clinician self-report

The sensitivity and specificity for clinical behaviours categorised as 'must do' and 'must not do' actions are presented in Figure 2 for one report that that used clinical vignettes to elicit clinician self-reported behaviour [11].

Sensitivities and specificities ranged from 0.47 to 0.95 and 0.40 to 0.80, respectively, for 'must do' and 'should do' behaviours, and from 0.20 to 0.70 and 0.45 to 0.90, respectively, for 'must not do' and 'should not do' behaviours (Figure 2). Positive (PPV) and negative (NPV) predictive values were also calculated for this study [11]. PPVs ranged from 0.17 (cold relief: physician required/should not do) to 0.89 (cold relief: recommend medication/should not do (Median = 0.42) (Figure 3). NPVs ranged from 0.50 (cold relief: physician required/should do) to 1.00 (cold relief: recommend medication/must not do), median = 0.80 (Figure 3).

Item-by-item comparisons evaluating clinician self-report were made by three further reports that used methods other than sensitivities and specificities [12-14]. Gerbert *et al. *(1986) [14] report 84% total agreement between clinician self-report and a video-recording of the consultation. Gerbert *et al. *(1988) [12] presented a kappa coefficient of 0.67 for the level of concordance between clinician self-report during interview and video-recording, and a total disagreement between these measures of 13%. Pbert *et al. *[13] made comparisons across measures for the detection of individual items using Cochrane's *Q *tests. These comparisons suggest that clinicians tended to over-report their behaviour on some items compared to audio-recording.

### The accuracy of clinician self-report

A ROC curve was plotted for the one study where both sensitivity and specificity could be calculated for several, 'must do/not do' and 'should do/not do' clinical actions [11] (Figure 4). Behaviours categorized as 'should not do' tended to group in the top left quadrant of the plot, tentatively suggesting that clinician's accurately report for such behaviours (*e.g.*, should not recommend medication for cold relief). Accuracy was poorer for behaviours categorized as 'must not do' and 'should do' (which tended to group in the bottom left quadrant of the plot) and behaviours categorized as 'must do' (which tended to fall into the top right quadrant of the plot).

### Studies combining items into summary scores

#### Patient report

One report that evaluated patient report and made item-by-item comparisons also combined items into summary scores [13]. Pbert *et al. *[13] calculated scores that represented the number of smoking advice intervention steps taken by a clinician during a patient consultation. The correlation of these scores between patient report and audio-recording was r = 0.67.

#### Medical record review

Three reports evaluating medical record review [15,17,18] presented summary percentage scores (65.6%, 54.0%, and 45.8%, respectively) that were consistently lower than scores reported by a standardised patient (76.2%, 68.0%, and 61.7%, respectively). One report [17] reported an overall correlation coefficient of r = 0.54 between summary scores relating to the management of commonly presenting outpatient conditions (Table 2).

#### Clinician self-report

Six reports evaluating clinician self-report calculated summary scores [11,13,15,16,18,19]. Different reports compared these self-reports to different direct measures.

One report [16] presented scores for the mean number of clinical actions performed by a group of clinicians as measured by each method in relation to the management of urinary tract infection (mean (SD) self-report = 9.88 (3.44), standardised patient report = 10.04 (3.37)). Rethans *et al. *[16] also presented subgroup means that suggest clinicians under-report their performance for 'obligatory' actions and over-report for less essential 'Intermediate' and 'superfluous' actions (Table 2). Two reports calculated the proportions for actions correctly performed; one in relation to the management of common outpatient conditions (% (SD) self-report = 71.0 (5.4), standardised patient report = 76.2 (7.2)) [18], and one in relation to the provision of preventive care advice (% (SD) self-report = 48.3 (14.4), standardised patient report = 61.7 (12.9)) [15]. Page *et al*. [11] present an overall total agreement of 66% between self-report and standardized patient report.

Three reports [11,13,19] present correlation coefficients of: 0.26 to 0.68 [11] for the relationship between performance on clinical vignettes and standardized patient reports; 0.21 for a global self-estimate of performance of hand hygiene actions with direct observation [19]; and 0.54 for clinician self-reported provision of smoking cessation counselling compared with audio-taped accounts of the consultation [13].

## Discussion

### Validity of the direct measures used

A problem in assessing any proxy measure of clinician performance is the validity of the direct measure itself as a true reflection of actual behaviour. Simulated patients (standardised patients) have been widely used in medical education, and there is an extensive literature to support their validity as a 'gold standard' method for measuring clinical behaviour [12,14,18]. Standardised patients require careful and detailed training in the clinical case they are to represent [20], and for those studies reviewed here that provide information about the training of standardised patients, this appears to have been adequate [20]. Three included studies assessed detection rates by clinicians, and reported these to be low. The six studies [10,11,15-18] that used simulated patients specify very precisely the characteristics of the cases presented to the clinicians. The other studies observed the clinicians' behaviour with actual patients and therefore had less control over the clinical situation in which behaviour was assessed, but are likely to be more generalisable to real-life clinical situations.

Direct observation using trained observers, audio- or video-recording are also methods that are commonly used as direct measures of clinical behaviour. However, one study [14] using video-recording of consultations found that relevant clinical detail – for example, assessment of symptoms and signs – was more frequently reported as having been done when measured by clinician self-report. Taken at face value, this may suggest over-reporting on behalf of clinicians. However, it is feasible that some aspects of the clinical assessment of symptoms and signs are performed non-verbally. In another study, the measurement of blood pressure was accurately recorded in the patient medical record but was not detected by the direct measure used (audio-recording) [7]. It is also plausible that, while we can expect that standardized patients may observe a clinician making an entry in a medical record, they could not accurately comment on the content of the entry. A further example of the limits of capture for direct measures can be seen in one of four reports that compared the direct measure of audio-recording with the proxy of medical record review [9]. This report found that while some clinical actions investigated (for example, the discussion of a diagnosis or drug name during a consultation with a patient) were not detected during evaluation of the audiotape session a diagnosis and the name and dosage of drugs prescribed had been recorded in medical records by the physician. As an aim of this report was to evaluate clinician communication with patients, the direct measure was valid as it gave an accurate account of what the physician did, and did not, communicate to the patient. However, audio-recording would lack validity as a direct measure for the making or documenting of a diagnosis and some related management decisions.

This suggests that there are very few gold standard, direct methods for assessing clinical performance – possibly only standardised patient methodology and participant observation – that can validly cover an extensive range of clinical actions, and that none can truly capture all aspects of behaviour. A direct measure can only be a valid gold standard for any given behaviour of interest, if it can reliably capture that behaviour.

### Validity of the proxy measures used

The accuracy of three proxy measures was reviewed: patient report, medical record review, and clinician self-report. These indirect measures were used by the included reports to estimate the performance of a wide range of clinical actions. The accuracy of each proxy measure varied across the clinical behaviours measured. Reports evaluating clinician self-report and patient-report also used different techniques to capture the measure of behaviour (*e.g.*, interview, self-completion questionnaire, patient vignettes).

### Patient report

Patient-report measures demonstrated greater accuracy than the other two proxy measures for reporting clinician performance, particularly with respect to counselling behaviours and routine procedures. A cautionary adjunct to this, however, is the finding of one study that the predictive validity of patient-reported information deteriorates markedly as the time between patient exposure to clinician behaviour and the timing of their recall of events increases [8]. Also, patient recall was found by another study to be significantly influenced by the duration of the advice and factors relating to relevancy, *i.e.*, advice provided during well-care consultations and the presence of a health behaviour-relevant diagnosis during an illness visit [6].

### Medical record review

Medical record review appeared to underestimate many aspects of clinician behaviour, particularly in the domain of patient counselling. Thus, our findings suggest that medical record review, in the outpatient setting, lacks validity as a general measure of clinician behaviour. However, there was evidence to suggest that the predictive ability of medical record review improves substantially for, but is restricted to, specific types of clinical action, for example, physical examination, the recording of drug dosages, and the ordering of laboratory tests. Medical records may therefore be a relatively low-cost and accessible proxy measure for these clinical behaviours. Medical records may also be advantageous in that they can be good 'history keepers' because they can store information from several consultations and a variety of conditions.

### Clinician self-report

The accuracy of clinician self-report as a measure of actual behaviour is harder to establish because different studies using different methods produced different outcomes. Also, none of the studies evaluating clinician report used appropriate statistical methods to summarise and/or report the relationship between the measures used.

Four reports that calculated summary scores of performance on vignettes appear to suggest that clinician's self-reported estimates of their behaviour were, overall, close to those generated by the direct measure. However, closer examination of the individual behaviours contributing to the overall summary scores by one of these studies [16] revealed that clinicians were overestimating their performance of some clinical actions and underestimating their performance of others, an observation lost in the summary score due to counterbalancing. Over- and underestimation was also tentatively suggested on the ROC plot for an additional study [11], albeit in a contrasting direction.

Of these two studies demonstrating over- and underestimation of self-reported behaviour, one provided clinicians with a closed-ended checklist of possible behaviours [11]. The second study used an open-ended response mode with responses coded later by an independent observer [16]. This may explain the conflicting outcomes of these two studies; because closed-ended checklists provide clinicians with an extensive list of possible actions, they may produce a cueing effect for them to select additional actions or act as a prompt to elicit knowledge about what they could, or should not do [21-23]. Such variation in the ability of vignettes to predict the occurrence of important behaviours that clinicians should or should not do undermines their validity. However, this may be a problem that can be overcome by careful and rigorous development of vignette cases and the method of their presentation [21].

Measures that use vignettes require clinicians to report their behaviour in the context of what they would do in a given clinical scenario. The remaining studies evaluating clinician self-report collected retrospective accounts of actual behaviour using either interview or questionnaire methods and report correlation coefficients and measures of 'total agreement' that suggest good agreement between measures. However, correlation is a measure of association, and a high correlation can effectively disguise important disagreement if there is a consistent bias in one measure [24]. A similar problem exists with the interpretation of 'total' or 'observed' agreement in that a large proportion of the agreement may be for behaviours that were reported by both measures as not performed, again disguising important deficits in a proxy measure to accurately detect actual performance [25].

### Review limitations

Many references reviewed were sourced from the reference lists of retrieved articles. We did not find a common terminology for describing written case simulations or proxy methods, and it is therefore possible that our database search was subsequently limited by this. A common terminology for measures would greatly facilitate research in this area. The literature search only covered up to August 2004; an update of this review could provide further useful information. A further limitation of this review is that we were not able to combine data due to the heterogeneity of the included reports. We tried to minimise publication bias by searching not only the peer-reviewed literature but also abstracts of conferences and unpublished theses. As we were unable to conduct a formal meta-analysis because of the heterogeneity in the designs, proxy measures, and summary statistics used in the included studies, we could not use conventional methods of assessing publication bias [26]. Nevertheless, the included studies presented various results – seven studies [5-7,9,11,14,17] presented a range of both positive and negative findings, six studies [8,10,12,13,15,18] presented positive findings only and one [16] presented only negative or inconclusive findings – suggesting that there is no apparent systematic tendency towards publication bias in the current review.

## Conclusion

In validating a proxy measure of clinical behaviour it is imperative that the direct measure for comparison is itself both reliable and valid. In some of the included reports the direct measure lacked validity. Only four studies were found that used appropriate statistical methods to compare measures. The validity of patient report and medical record review varied widely across a number of clinical actions but was high for some specific clinical actions. The evidence for the validity of clinician self-report is inconclusive.

Two recent systematic reviews evaluated the efficacy of social cognitive models of behaviour in explaining clinical performance [3,27]. Both reviews found that the relationship between clinicians' self-reported intention and their behaviour is not perfect (maximum R^2 ^reported was 0.44 [27]), and that the strength of the relationship often varied depending on the method used to measure their behaviour. The current review supports the notion that at least some of the discrepancy between intentions and behaviour can be explained by error originating from unreliable measures of behaviour.

Valid measures of clinical behaviour are of fundamental importance to accurately identify gaps in care delivery, to continuous improvement of quality of care, and ultimately to improved patient care. However, the evidence base for three commonly used proxy measures of clinicians' behaviour is very limited. Further research needs to establish the scope of capture for a range of both direct and indirect measures of clinical behaviour and the potential for using a combination of proxy measures to obtain an all round picture of clinical behaviour.

## Competing interests

The authors declare that they have no competing interests.

## Authors' contributions

All authors contributed to the conception and design and analysis of the study and approved the submitted draft. MPE, JJF, EK SH and HD reviewed the articles and abstracted the data.

## Supplementary Material

Additional file 1 **Characteristics of included studies**. Detailed description of the characteristics of all studies included in the review. Click here for file

Additional file 2 **Results presented by studies included in the review**. Detail of the samples, analyses and outcomes presented by studies included in the review. Click here for file

## References

[B1] The Information Centre Quality and Outcomes Framework for GP practices. http://www.ic.nhs.uk/.

[B2] Department of Health (2003). New GMS Contract 2003. Investing in general practice.

[B3] Eccles MP, Hrisos S, Francis J, Kaner EF, Dickinson HO, Beyer F, Johnston M (2006). Do self-reported intentions predict clinicians' behaviour: a systematic review. Implement Sci.

[B4] Streiner DL, Norman GR (2003). Health Measurement Scales: a practical guide to their development and use.

[B5] Stange KC, Zyzanski SJ, Smith TF, Kelly R, Langa DM, Flocke SA, Jaen CR (1998). How valid are medical records and patient questionnaires for physician profiling and health services research? A comparison with direct observation of patients visits. Medical Care.

[B6] Flocke SA, Stange KC (2004). Direct observation and patient recall of health behavior advice. Prev Med.

[B7] Wilson A, McDonald P (1994). Comparison of patient questionnaire, medical record, and audio tape in assessment of health promotion in general practice consultations. Source. BMJ.

[B8] Ward J, Sanson-Fisher R (1996). Accuracy of patient recall of opportunistic smoking cessation advice in general practice. Tobacco Control.

[B9] Zuckerman ZE, Starfield B, Hochreiter C, Kovasznay B (1975). Validating the content of pediatric outpatient medical records by means of tape-recording doctor-patient encounters. Pediatrics.

[B10] Luck J, Peabody JW, Dresselhaus TR, Lee M, Glassman P (2000). How well does chart abstraction measure quality? A prospective comparison of standardized patients with the medical record. American Journal of Medicine.

[B11] Page GG, Fielding DW (1980). Performance on PMPs and performance in practice: are they related?.

[B12] Gerbert B, Stone G, Stulbarg M, Gullion DS, Greenfield S (1988). Agreement among physician assessment methods. Searching for the truth among fallible methods. Medical Care.

[B13] Pbert L, Adams A, Quirk M, Herbert JR, Ockene JK, Luippold RS (1999). The patient exit interview as an assessment of physician-delivered smoking intervention: a validation study. Health Psychol.

[B14] Gerbert B, Hargreaves WA (1986). Measuring physician behavior. Medical Care.

[B15] Dresselhaus TR, Peabody JW, Lee M, Wang MM, Luck J (2000). Measuring compliance with preventive care guidelines: standardized patients, clinical vignettes, and the medical record. Journal of General Internal Medicine.

[B16] Rethans JJ, van Boven CPA (1987). Simulated patients in general practice: a different look at the consultation. British Medical Journal.

[B17] Rethans JJ, Martin E, Metsemakers J (1994). To what extent do clinical notes by general practitioners reflect actual medical performance? A study using simulated patients. British Journal of General Practice.

[B18] Peabody JW, Luck J, Glassman P, Dresselhaus TR, Lee M (2000). Comparison of vignettes, standardized patients, and chart abstraction: a prospective validation study of 3 methods for measuring quality. JAMA.

[B19] O'Boyle C, Henly S, Larson E (2001). Understanding adherence to hand hygiene recommendations: the theory of planned behavior. Am J Infect Control.

[B20] Buellens J, Rethans JJ, Goedhuys J, Buntinx F (1997). The use of standardised patients in research in general practice. Family Practice.

[B21] Peabody JW, Luck J, Glassman P, Jain S, Hansen J, Spell M (2004). Measuring the quality of physician practice by using clinical vignettes: a prospective validation study. Annals of Internal Medicine.

[B22] Spies T, Mokkink H, De Vries Robbe P, Grol R (2004). Which data source in clinical performance assessment? A pilot study comparing self-recording with patient records and observation. International Journal for Quality in Health Care.

[B23] Jones TV, Gerrity MS, Earp J (1990). Written case simulations: do they predict physicians' behaviour?. Journal of Clinical Epidemiology.

[B24] Chia KS (2000). Association or Agreement?. Annals Academy of Medicine Singapore.

[B25] Hripcsak G, Heitjan DF (2002). Measuring agreement in medical informatics reliability studies. Journal of Biomedical Informatics.

[B26] Egger M, Davey Smith G, Altman DG, (Eds) (2001). Investigating and dealing with publication and other biases Chapter 11 in Systematic reviews in health care: meta-analysis in context.

[B27] Godin G, Belanger-Gravel A, Eccles MP, Grimshaw J (2008). Healthcare professionals' intentions and behaviours: A systematic review of studies based on social cognitive theories. Implementation Science.

