# Supplementary material for: Are there valid proxy measures of clinical behaviour? a systematic review
Source: Implement Sci. 2009 Jul 3;4:37. doi: 10.1186/1748-5908-4-37 (PMC2713194; doi:10.1186/1748-5908-4-37)
Supplement: Additional file 1 — Characteristics of included studies. Detailed description of the characteristics of all studies included in the review. [file 1748-5908-4-37-S1.doc]

|  | **Aims/Hypothesis/RQ Design** | **Setting and Participants** | **Behaviour/s and how scored and/or summarised** | **Direct measure**  **(Actual behaviour)** | **Proxy measure**  **(proxy behaviour)** |
| --- | --- | --- | --- | --- | --- |
| **5. Stange 1998 USA** | Aims: This study was designed to determine the optimal non-observational method of measuring the delivery of outpatient medical services. Aimed to examine the inter-rater reliability (IRR) and validity of the commonly used and relatively inexpensive medical record review and patient questionnaire methods compared to direct observation of the outpatient visit.  Design: Non-experimental, prospective | Family practice setting. Convenience sample of family physicians who were members of the Ohio Academy of FPs and whose practice was within a 50 mile radius of Cleveland and Youngstown.  Excluded if: physician not practicing in family practice setting or were a full time academic physician. However, 30 members of the faculty of the NE Ohio Universities Colleges of Medicine were included. Consecutive patients attending the clinic on the day were recruited to the study.  Temporal specification:  Summer of 1994 | Behaviour: Delivery of out-patient medical services, particularly preventive services.  Scoring method: Direct observation checklist: Davis Observation Code which categorises time use during every 15-second interval of each patient visit into 20 different behavioural categories. Used to code all measures according to whether or not particular services were delivered during the observed visit.  Psychometrics: kappa for IRR ranged from 0.39 to 1.00 across a number of services delivered. | Consultations were directly observed by a research nurse who completed a series of checklists. | Medical record: abstracted by a research nurse (79% by nurse different to observer).  Patient exit questionnaire. |
| **6. Flocke 2004**  **USA** | Aim: Using direct observation of 2,670 adult patient encounters, to report the rate of patient recall of nine preventive health behaviours using direct observation and patient self-report. Also, investigation of visit and patient factors associated with recall of advice.  Design: Questionnaire, retrospective | Primary Care. Family physicians in NW Ohio.  Included if: NR for doctors. Consecutive patients consulting during the two observation days. Over the age of 18 years.  Temporal specification:  October 1994 through August 1995. | Behaviour: Health promotion advice during consultation  Scoring method: Direct observation code used. Classifies visit into 20 different non-mutually exclusive behaviour categories that are assessed every 20 seconds during observed visits.  Psychometrics: IRR for four teams of two research nurses was ‘very good’. | Direct observation of provision of health behaviour advice on: smoking, exercise, diet, alcohol use, illicit substance use, sun exposure, seatbelt use, HIV prevention, STD prevention. | Patient-report: Patient recall measured by self-report questionnaire. Questionnaire given to patient at end of visit, to take home if necessary. |
| **7. Wilson 1994 UK** | Aims: To determine what proportion of health promotion activities reported by the patient as recorded in the general practice notes, and to compare these methods of assessing health promotion with audio tape recording.  Design: Non-experimental, (patient) questionnaire prospective | Primary care. General practitioners (GPs) from 10 general practices in Nottinghamshire. Minumum of two non-randomly selected consultations for each GP were audiotaped.  Included if: consultation was with adult patient (aged 17 and over). Patients who did not declare their smoking habits were excluded from analysis.  Temporal specification:  Data collection took place from 1987 to 1989 | Behaviour: Health promotion advice during consultation  Scoring method: Patient questionnaire: ‘did the doctor talk to you about smoking or alcohol or take your blood pressure?’ Responses format was Y/N.  Medical records and audiotapes: Any mention by a doctor of smoking, alcohol, diet or exercise counted as positive.  Psychometrics: Medical records: IRR between three pairs of researchers was 0.96 to 1.00 for BP, and between 0.89 to 1.00 for smoking and alcohol advice.  Audiotape: BP = 0.79 TO 0.94; Alcohol and smoking = 0.84 to 0.97 for 44 audiotaped consultations. | Audio tape of consultations | Patient exit questionnaire  Medical record: abstracted by research assistant. |
| **8. Ward 1996 Australia** | Aim: to determine the accuracy of patient recall of a question about smoking in a specified consultation in general practice and the accuracy of smokers’ recall of advice to stop smoking.  Design: Non-experimental, questionnaire | Primary care.  Post-graduate trainees in GP training practices (and consecutive samples of their patients aged 16 to 65) in New South Wales.  Temporal specification:  NR  Included if: Consenting patients; trainees’ first ever experience of three months in supervised general practice. | Behaviour: Provision of smoking cessation advice  Scoring method: Four coders rated tapes for a question about smoking or advice to stop smoking. Response format was occurred or not occurred.  Patient report — questionnaire asked if had been asked if they smoked, and for smokers only, if they had been given advice to stop smoking. Response format yes, no can’t recall (treated as missing data). | Audiotaped consultations | Patient-report: Self-administered questionnaire mailed to patients within two days of consultation. |
| **9.Zuckerman**  **1975**  **USA** | Aims: To appraise the extent to which the record serves to document adequately the content of verbal communications between practitioners and patients.  RQs: to what extent are actual communication behaviours recorded in case notes?  Design: Non-experimental, prospective | University Medical Centre Clinic  Paediatricians serving an inner-city population (assumed to be Baltimore, Maryland). All three staff physicians were sampled. Included if: completed paediatric training; working at clinic for more than six months.  Temporal specification: two Week period in august 1973 | Behaviour: verbal communication with patients  Scoring method: Tapes and medical records were reviewed and categorised according to the presence or absence of a variety of items performed (n = 8) and a range of historical items (n = 7) which were present in both the tape and record, present in the record but not the tape, present in the tape but not the record, present in both but significantly different in content (not merely terminology), or absent from both tape and record.  Response format: Yes/No  Psychometrics: NR | Audio recording of consultation.  For each participant (paediatrician) one consultation in the morning and one in the afternoon were audio-taped, each with the first available patient.  Events were sampled once in am and once in pm during a two-week period using first available patient <13years old, but in terms of clinicians, n = 3). | Medical record: Case note review  Timing: Concurrent. Records relating to a visit and a tape recording of that consultation.  Patient interviews immediately after consultation |
| **10. Luck 2000**  **USA** | Aim: We prospectively evaluated the validity of chart abstraction by directly comparing it with the gold standard of reports by standardised patients.  Design: Non-experimental, prospective trial. | General internal medicine primary care outpatient clinics at two Veteran Affairs medical centres (West Los Angeles and San Diego, California). All primary care staff physicians, faculty and residents  Excluded if: NR  Temporal specification: Between Feb and August 1997 | Behaviour: Management of four common outpatient conditions: low back pain, diabetes mellitus (DM), chronic obstructive pulmonary disease (COPD), coronary artery disease (CAD).  Scoring method: Checklist of explicit quality measurement criteria. The percentage of necessary criteria that were correct was determined as the number of correct responses divided by the number of potential quality criteria.  Psychometrics: NR | SP representing one simple, and one complex case for of four common outpatient conditions. | Medical record: abstracted by a trained nurse.  Timing: Medical records were abstracted immediately following the SP visit. SP completed checklist immediately following their visit. |
| **11. Page**  **1988**  **Canada** | Aim: Study was conducted to test the criterion validity of a set of patient management problems (PMPs) through a direct behaviour by behaviour comparison of performance on the PMPs and performance in the practice setting.  Design: Experimental using non-equivalent control group. The continuing education course participants were designated the experimental group. | Community/ Pharmacists  Included if: not specified  Temporal specification: Patient Management Problems (PMP) and In-Store Assessment Problems (ISAP) were administered before and after the course to both experimental and control groups. Pre-course ISAPs were administered five to six weeks before matched PMP. Post-course ISAPs were administered one to three weeks after matched PMP. | Behaviour: Advice given in response to two patient requests: ‘What is the strongest pain reliever I may purchase without a prescription?’ and ‘What have you got for a cold?’  Scoring method: SPs completed a behavioural checklist rated on a weighted five-point scale: must do (5) to must not do (1). Scores on the PMPs were calculated by summing the weight of the items selected. Scores on ISAPs were the sum of weights of behaviours exhibited by pharmacist.  Psychometrics: 0.76 for SP ratings across four PMPs. | SP — role playing actor presenting a primary care request to pharmacist in own store (referred to by authors as ‘In-store Assessment Problem or ISAP). | Vignette: Written simulations of patient management problems (PMPs) identical to ISAP presented by SP. four matched cases were developed.  Timing: |
| **12. Gerbert 1988 USA** | Aim: To determine the convergent validity of four methods of physician assessment in relation to medication regimens for patients with COPD.  Design: Non-experimental | Primary care. Physicians serving six counties in California, who volunteered to take part in a study assessing quality of care.  Adult patients with COPD, selected by their participating physician  Included if: Doctors could provide information for two or more patients. Patients with a diagnosis of COPD. | Behaviour: Prescribing of medications for patients with COPD.  Scoring method: percentage of patients on medication for COPD | Video recording of consultation | Physician interview, patient interview, chart audit |
| **13. Pbert**  **1999**  **USA** | Aim: To assess how well patients’ perceptions (of smoking cessation counselling) conform to a criterion measure of an independent judge’s evaluation of an audio-tape of the physician-patient interaction. Primary interest is comparison of patient exit interviews (PEI) with audio recordings of consultations  Design: Non-experimental, prospective, Interview | Primary Care  Primary care physicians in University medical centre in Massachusetts.  Convenience sample of 13 attending physicians and their patients. Patients approached to take part by their physician.  Included if: attending physician; patients eligible if aged 18 to 35 and current smokers (at least one puff in last seven days).  All physicians had received training in the use of a brief provider-delivered, patient-centred counselling intervention for smokers.  Temporal specification: six -month period | Behaviour: provision of smoking cessation counselling  Scoring method: Patient Exit Interview (PEI , Physician self-report exit interview (MDEI) responses scored as ‘yes’ or ‘no’, and audiotape as ‘present’ or ‘absent’. Overall score ranged from 0 to 10. Scoring instrument was identical to the PEI and MDEI in content and scoring of 15 counselling steps (measures of behaviour) related to smoking cessation counselling.  Pyschometrics: NR | Audio recording of consultation  Audio-tapes were scored independently by two judges, blind to PEI or MDEI content for individual tapes. | PEI: in person interview with research assistant.  MDEI  Timing: MDEI completed at end of consultation (immediately following patient visit). PEI completed immediately following patient visit |
| **14. Gerbert 1986 USA** | Aim: To examine inter-rater reliability of four methods for measuring physician behaviour and their content coverage and convergent validity  Design: Non-experimental, interview. | Primary care. Convenience sample of physicians who responded to a mailed invitation to participate in a study. Adult patients with COPD, selected by their participating physician  Included if: NR for doctors. Patients with a diagnosis of COPD.  Temporal specification:  All measures related to the same consultation | Behaviour: Management of COPD, symptoms, signs, tests, treatments, patient education.  Scoring method: Specification of behaviour according to a ‘criteria map’ that defines adequate physician performance in treatment of COPD. Specifies 75 items that were coded from videotapes and patient charts and assessed in physician and patient interviews. Of the 75 items, 20 were selected for their importance in COPD management and clarity of definition. Items coded as present or absent from consultation.  Psychometrics: ‘Excellent’ inter-rater agreement (Kappas >0.80 for all methods). Content validity assessed and found to be good for the two interview methods. | Video recording of consultation | Medical record review  Physician interview  Patient interview |
| **15. Dresselhaus 2000 USA** | Aim: To determine how accurately preventive care reported in the medical record reflects actual physicians’ practice or competence  Design: Non-experimental, prospective trial. | Primary care, general internal medicine outpatient clinics at two Veteran Affairs medical centres (West Los Angeles and San Diego, California).  All primary care staff physicians, faculty and residents excluded if: NR  Temporal specification:  December 1996 and August 1997 | Behaviour: Management of four common outpatient conditions: low back pain, DM, COPD, CAD.  Scoring method: Identical criteria were used in each method as explicit items on which to score provider responses for each of the eight cases. ‘Quality scores’ were generated from Checklist of preventive care scoring criteria derived from national guidelines and a modified Delphi technique, generating seven preventive care items: tobacco screening, advice regarding smoking cessation (all cases were smokers), past performance of prevention measures (immunization and cancer screening), alcohol screening, diet evaluation, assessment of exercise, and counselling on physical activity.  Six to seven preventive care items were scored for each of the 160 physician-patient visits. Percentile scores were generated for each method (%of visits in which preventive practice occurred). Also calculated the marginal percentile improvement of SP checklists and vignettes over the chart abstraction score alone.  The face validity of the results was assessed against a random sample of VA patients who were surveyed for patient report of delivery of preventive care items. | SP representing one simple, and one complex case for four common outpatient conditions.  SP visit occurred three months post consent to diminish recall of study | Vignette: Eight detailed scenarios representing one simple, and one complex case for each of four common outpatient conditions  Medical record: abstracted by a trained nurse.  Timing: Vignettes completed by participating physicians after SPs had been seen. Medical records were abstracted immediately following the SP visit. SP completed checklist immediately following their visit. |
| **16. Rethans 1987**  **Netherlands** | Aims: 1. To investigate the possibility of using trained SP to collect data about the reality of the consultation. 2. To measure the difference between the performance of GPs when dealing with the SP (what the doctor does) and the actions that the same GP said they would take when dealing with a written problem about a similar patient (what the doctor says he would do).  Hypothesis: GPs would do less in a consultation with the simulated patient than they would have indicated in their answers to the written problem (identical patient scenario)  Design: Non-experimental, prospective | Primary Care  All 378 GPs working in the same county as authors’ University (Maastricht) were sampled.  Included if: employed a secretary still connected with the university’s school for medical secretaries and returned written simulation.  Excluded if: responded to information about study by saying didn’t want to participate; connected with Dept of general practice of the university; active withdrawal; practice too far away; detected SP.  Temporal specification: Not Reported (NR) | Behaviour: Management of Range of behaviours relating to Urinary Tract Infection (UTI) covering: history taking; physical exam; instructions to patient; treatment and follow-up (see Table 1), coded as obligatory, intermediate or superfluous.  Scoring method: Performance using both methods was scored according to an existing consensus standard on urinary tract infection developed at the University of Nijmegen. | Standardised SP  Psychometrics: Validity of SP reports carefully validated against judgements of three independent doctors | Vignette: Written simulation, identical match to SP. Case simulations were used as basis for the training of SPs.  Timing: two months following visit by SP |
| **17. Rethans 1994**  **Netherlands** | Aim: this study set out to examine the extent to which clinical notes in medical records of general practice consultations reflected doctors’ actual performance during consultations.  Design: Non-experimental, prospective | Primary care GPs. Sampling strategy reported elsewhere.  Included if: NR  Temporal specification: During 1989 | Behaviour: management of four commonly presenting conditions; tension headache; acute diarrhoea; pain in the shoulder; check-up for non-insulin dependent diabetes.  Scoring method: ‘Extensive’ checklist based on accepted standards of care was completed by SP immediately following consultation with GP. The standards described ‘essential actions’ and ‘immediate actions’ (four standards — each included between 25 and 36 actions).  Summary statistic: A ‘content score’ was calculated as a measure of agreement between actions which had been recorded and actions which could have been recorded/actions performed and recorded and performed but not recorded. A high content score reflected a consultation that had been recorded well. | SP representing complaints presented for: tension headache, acute diarrhoea, pain in the shoulder, check up for non-insulin dependent diabetes. | Medical record abstraction (examined two years later).  Psychometrics: Agreement between raters of medical records: kappa=0.93 |
| **18. Peabody 2000**  **USA** | Aim: To validate clinical vignettes as a method for measuring the competence of physicians and the quality of their actual practice.  Design: Non-experimental, prospective trial. | General internal medicine primary care out-patient clinics at two Veteran Affairs medical centres (West Los Angeles and San Diego, California).  All primary care staff physicians, faculty and residents  Excluded if: Interns  Temporal specification: Feb though July 1997 | Behaviour: Management of four common outpatient conditions: low back pain, DM, COPD, CAD.  Scoring method: Identical criteria were used in each method as explicit items on which to score provider responses for each of the eight cases. Scores’ were generated from SP responses to a closed-ended post-interview questionnaire. Medical record abstraction and vignette scores were derived from scoring forms that contained the same criteria. Raw item scores for each method were aggregated into category scores for that method. These weighted scores, which averaged 21 categories per case, were totalled and divided by the total possible score, generating a percentage correct score for each physician-case combination.  Psychometrics: NR | SP representing one simple, and one complex case for four common outpatient conditions. | Vignette: Eight detailed scenarios representing one simple, and one complex case for each of four common outpatient conditions.  Medical records: abstracted by a trained nurse.  Timing: Vignettes completed by participating physicians ‘several’ weeks after SPs had been seen. Medical records were abstracted immediately following the SP visit. SP completed checklist immediately following their visit. |
| **19. O’Boyle 2001 USA** | Aim: (2). To describe relationships among variables from the TPB, self-reported adherence, observed adherence and level of activity in the nursing unit.  Design: Non-experimental, Questionnaire, prospective | Hospital intensive care and post-intensive care unit.  Nursing staff working in medical/surgical intensive care units and the associated post-intensive care units in four metropolitan teaching hospitals in ‘midwest’ USA. Units were selected to include patient populations with comparable nursing care requirements across hospitals.  Included if: staff and charge nurses provided direct patient care; employed for at least six months; worked at least one day per week on average.  Temporal specification:  July 1996 to October 1997 | Behaviour: Hand hygiene  Measure: Hand hygiene events were recorded when nurses washed their hands with the handwashing agent and water using the ‘Handwashing Assessment Inventory’ protocol. Observers recorded the number of indications for handwashing and the number of times nurses washed their hands when the indications arose.  Adherence score was for seven indications and overall. Adherence score was (n occasions when hands were washed / n occasions when hand washing was indicated).  Psychometrics: IRR for two observers from pilot was 0.94 and 0.98 | Direct observation: Participants were observed for two hours or until 10 indications for handwashing had occurred. | Self-report: percentage of time (0 to 100) that the nurse practiced hand hygiene when indicated (before care; when care was interrupted; between patients; before performing an invasive procedure; after contact with contaminated material and before beginning a clean procedure on the same patient; after removal of gloves; after direct contact with body fluids; before touching own mouth, nose, eyes, and face with contaminated hands). |
