# Supplementary material for: Are there valid proxy measures of clinical behaviour? a systematic review
Source: Implement Sci. 2009 Jul 3;4:37. doi: 10.1186/1748-5908-4-37 (PMC2713194; doi:10.1186/1748-5908-4-37)
Supplement: Additional file 2 — Results presented by studies included in the review. Detail of the samples, analyses and outcomes presented by studies included in the review. [file 1748-5908-4-37-S2.doc]

| Study | Sample | Comparison/Analysis | Outcome |
| --- | --- | --- | --- |
| 5. Stange 1998 USA | Approached: 531  Enrolled: 138  Analysed: 128  Percentage analyzed: 93  Mean age (yrs): 43 (7.6)  Percentage Male: 72 | Item by Item.  Sensitivity and specificity. Kappa for IRR. T-tests and Wilcoxon and chi-square to establish representativeness of samples. Kappa coefficients to analyse the concordance between DIRECT MEASURE and proxy measures. Medical records were available for 99% of observed visits. Patient exit questionnaires were available for 74% visits. To improve the stability of estimates of sensitivity, specificity and kappa, data were presented only for services with at least 30 observations | Medical Records:  Sensitivity = 8% (diet advice) to 92% (lab tests) Specificity = 83% (social history) to 100% (counselling services, physical exam, lab tests)  Patient Report:  Sensitivity = 17% (mammogram) to 89% (pap test)  Sensitivity = 85% (in-office referral) to 99% (immunisation)  Concordance of observed behaviour with:  Medical record review: kappa = 0.12 to 0.92 for 79 comparisons (n = 30 to 4,454)  Patient report: kappa = 0.03 to 0.86 for 53 comparisons (n = 32 to 2,024) |
| 6. Flocke 2004  USA | Approached: doctors NR, 4,994 patients. 4,454 patients agreed to have visit observed  Enrolled: 138 doctors in 84 practices  Analysed: 128 GPs. 2,670 patient questionnaires/observed consultations  Percentage analyzed: 93 GPs/60 consultations  Mean age (yrs): 52  Percentage male: 35 | Item by Item.  Sensitivity. Logistic regression. Percentage estimates to measure recall. | Sensitivity = 11% (substance use) to 76% (smoking cessation)  Health behaviour advice (observed n (%); patient recalled n (%)):  Smoking cessation: 154 (32); 117 (76)  Exercise: 603 (23); 260 (43)  Diet: 557 (21); 247 (44)  Smoking: 289 (11); 151 (52)  Alcohol: 250 (9); 73 (29) |
| 7. Wilson 1994 UK | Approached: NR  Enrolled:16  Analysed: 16 GPs from 10 practices/1,075 consultaions  Percentage analyzed: 100 GPs/72 consultations  Mean age (yrs): NR  Percentage male: NR | Item by Item.  Sensitivity. Inter-rater reliability, cohen’s kappa. Percent agreement between DIRECT MEASURE and proxy measures. The number of missing values varied between questions. Audio tape and patient questionnaires available for 335 consultations. Audiotape and medical notes available for 516 consultations. | Medical records:  Sensitivity = 31%, Specificity = 99% (smoking)  Sensitivity = 29%, Specificity = 100% (alcohol)  Sensitivity = 83%, Specificity = 93% (blood pressure)  Patient report:  Sensitivity = 74%, Specificity = 94% (smoking)  Sensitivity = 75%, Specificity = 94% (alcohol)  Sensitivity = 100%, Specificity = 90% (blood pressure)  Percent agreement between direct measure and:  Medical notes:  Smoking = 45.5  Alcohol = 28.6  BP = 83.3  Patient report:  Smoking = 81.8  Alcohol = 75.0  BP = 100  Reports same for agreement between proxy measures |
| 8. Ward 1996 Australia | Approached: 41 trainees  Refused: 7  Enrolled: 34 trainees  Analysed: 34  Percentage analyzed: 100  Median age (yrs): 27 (range 25 to 31)  Percentage male: 56 | Item by Item.  Sensitivity and specificity of patient recall as dichotomous variables calculated from two-by-two tables by comparing responses from patient questionnaires against audiotapes. 1,500 patients agreed to audiotaping; 1,362 usable tapes; 1,209 returned questionnaire. 1,075 matched tape/questionnaire; 451 smokers  Psychometrics: kappa for smoking status = 0.94; advice to stop = 0.74 | Q item ‘Were you asked about smoking?’  Sensitivity = 93%  Specificity = 79%  Q item ‘Were you given advice to quit?’  Sensitivity = 92%  Specificity = 82%  Question about smoking occurred in 29% consultations: advice to quit in 14% consultations with smokers. |
| 9. Zuckerman  1975  USA | Approached: 3 physicians  Enrolled: 3  Analysed: 3 physicians/51 consultations  Percentage analysed: 100  Mean age (yrs): NR  Percentage male: NR | Item by item  Concordance (presence/absence) between tape recordings and medical records of a variety of clinical actions.  No adjustment for clustering | % Concordance for seven historical items (range):  Actions present in both conditions (2% to 96%)  Actions present on tape, absent on record (0% to 51%)  Actions on absent on tape, present on record (0 to 4%)  Actions absent on both tape and record (0 to 86%)  Percentage concordance for eight diagnosis and management items (range):  Actions present in both conditions (0% to 67%)  Actions present on tape, absent on record (0% to 52%)  Actions on absent on tape, present on record (0 to 66%)  Actions absent on both tape and record (3 to 92%) |
| 10. Luck 2000  USA | Approached: 101 physicians  Not consented: 3  Enrolled: Randomly selected: 20 (10 physicians from each medical centre: 6 Faculty physicians, 7 third-year and 7 second-year residents)  Analysed: 20 physicians and 160 medical record scores (8 cases each)  Percentage analysed: 100  Mean age (yrs): NR  Percentage male: NR | Item by Item and Summary scores. Examined main effect using ANOVA (four-way). Sub-analyses assessed case effects; site and provider effects; domains; cuing and detection effects. Also evaluated the sensitivity and specificity of chart abstraction for categories of ‘necessary’ and ‘unnecessary’ behaviours. Controlled for case-mix and abstractor variability. | For necessary care the overall sensitivity was 70% and specificity was 81%. For unnecessary care sensitivity was 65% and specificity was 64%.  Summary scores (% correct responses (SD)):  All conditions:  SP = 68.0 (9.0)  Medical records = 54.0 (9.0)  ANOVA p-value: p<0.0001  Summary scores also presented for each of four conditions: SP scores were consistently higher than medical records.  ANOVA p-value:  LBP: p<0.0001  COPD: p<0.0001  DM: p<0.0001  CAD: p<0.0001 |
| 11. Page  1988  Canada | Approached: 30 pharmacists  Enrolled: 30  Analysed: 30 pharmacists, 58* observations on matched PMPs and ISAPs  *two pharmacists were not assessed on PMP or ISAP at baseline  Percentage analysed: 100  Mean age (yrs): NR  Percentage male: NR | Item by Item and Summary scores.  Pearson product moment correlations | PMP/ISAP paired case:   1. r = 0.56 (p<0.05) 2. r = 0.37 (p = ns) 3. r = 0.68 (p<0.05) 4. r = 0.26 (p = ns)   Agreement: Four PMPs used in this study were shown to possess content, construct and criterion validity to the extent of 0.66 agreement on behaviours common to both PMPs and ISAPs.  For each case, more behaviours exhibited on PMP than on ISAP: 18%; 15%; 25% and 19% respectively; Overall = 18%. |
| 12. Gerbert 1988 USA | Approached: 2,600  Analysed: 63 Physicians responded, 197 consultations observed  Percentage analysed: 100 (consultations)  Mean age (yrs): Patient 60.7, range 16 to 88. Physician NR  Percentage male: 90 (physicians) | Item by Item  Concordance between different methods using Kappa coefficient | Kappa coefficient for concordance of proxy measures for medication regimens with Direct measure (video recording of consultation):  Physician interview = 0.67  Patient interview = 0.50  Chart audit = 0.54  Agreement between proxy measures also reported |
| 13. Pbert  1999  USA | Approached: 13 physicians. 154 eligible patients identified through screening  Excluded: one physician refused; 46 patients due to: lack of time, physician forgot to audiotape, patient declined participation, patient not available due to immediate referral or tape recorder malfunction.  Enrolled:12  Analysed: 12 Physicians, 108 patients  Percentage analysed: 92 physicians, 70 patients  Mean age (yrs): 38 physicians, 42 patients  Percentage male: 100 physicians; 42 patients  %Caucasian: 92 physicians, 95 patients | Item by Item and Summary scores. Pearson product-moment correlation. Chi-square tests of proportions. Cochran’s Q test and kappa for agreement analysis. Multivariate logistic regression. Analysis of variance on PEI score with physician ID fit as a categorical predictor; examined differences between tape and patient assessment fitting physician ID as fixed effect; examined difference in tape derived mean by patient with average of physician averages. Mean number of nine patients per physician, maximum 20, most had 3 to 10. Examined possible effect of clustering for PEI. | Physician (Audio-recording)  Mean score: 6.3 (12.1)  Median: 8.0  Patient (PEI)  Mean score: 7.7 (1.7)  Median: 8.0  Physician (MDEI)  Mean score: 7.0 (2.2)  Median: 7.0  Mean % across 15 steps was 43.5% and 46.7% for average of physician average scores  Correlation co-efficient:  PEI and Audio-tape: r = 0.67 (p<0.0001)  PEI and MDEI: r = 0.67 (p<0.0001)  MDEI and Audio-tape: r = 0.77 (p<0.0001)  Clustering had minimal effect on results |
| 14. Gerbert 1986 USA | Approached: NR  Enrolled: 63 Physicians Analysed: 63 Physicians responded, 192 consultations observed  Percentage analysed: 100  Mean age (yrs): Patient 60.7, range 16 to 88. Physician NR  Percentage male: 90 (physicians) | Item by Item  Descriptive – pair-wise median percentage agreement between four assessment methods (Convergent Validity). Missing data: Measured 75 items but only presented the analysis of 20, chosen for their importance in COPD management and their clarity of definition in all four methods. | Median percentage agreement with Direct measure (video recording of consultation) across items within categories:  All categories:  Physician interview = 0.84  Patient interview = 0.86  Chart audit = 0.88  Agreement for each category also reported.  Agreement between proxy measures also reported |
| 15. Dresselhaus 2000 USA | Approached: 101 physicians  Not consented: Three  Enrolled: Randomly selected: 20 (10 physicians from each medical centre: six faculty physicians, seven third-year and seven second-year residents)  Analysed: 20 physicians and 160 medical record scores (8 cases each)  Percentage analysed: 100  Mean age (yrs): NR  Percentage male: NR | Item by Item and Summary scores.  Three-way ANOVA, which accounted for the design of the experiment; two-way comparison between methods using Student’s Neuman-Keuls method, which accommodates multiple comparisons and adjusts the pairwise significance accordingly. One-way ANOVA to compare chart to chart + SP to chart + SP + Vignette. Sub-analysed significant main effects using ANOVA and Neuman-Keuls comparing scores by site and training level of physician. Difference between mean SP and Veteran Affairs patient survey scores was examined using two-tailed t-test. | Preventive care score (Mean (SD)):  All care items:  SP = 45.8 (14.4)  Medical records = 61.7 (12.9)  Vignettes = 48.3 (10.4)  Three-way ANOVA p-value:  p<0.01  %difference (SP-chart) = 15.9  Scores also presented for each preventive care item (n = 7): three-way ANOVA p-value:  Tobacco screening: p<0.36  Alcohol screening: p<0.11  Diet evaluation: p = 0.02  All others: p<0.01 |
| 16. Rethans 1987  Netherlands | Approached: 378 GPs  Excluded: 323  309 did not fit inclusion criteria  14 had connections with Dept of General Practice or were too far away  Enrolled: 55  Three declined participation  Four not visited by SP  21 did not return scenario  Two detected SP  Analysed: 25  Percentage analysed: 46  Mean age (yrs): NR  Percentage male: NR | Summary scores  Two-tailed McNemar test for paired data with p<0.05  Student’s T-test for paired data to compare the total number of actions scored under both conditions with p<0.05.  Comparisons made on 24 obligatory, intermediate, and superfluous items | Significant differences in performance reported for 4/24 items, (N (%) GPs performing specified action, *p<0.05, **p<0.0005):  Category: History  Item 1*: Frequency  SP = 24/25 (96)  Vignette = 18/25 (72)  Category: Phys Exam  Item 2*: Superfluous action  SP = 5/25 (20)  Vignette = 13/25 (52)  Category: Instructions to patients  Item 3**: Explain diagnosis  SP = 23/25 (92)  Vignette = 6/25 (24)  Item 4*: Explain prognosis  SP = 12/25 (48)  Vignette = 2/25 (8)  Mean (SD) and range of actions performed, *p<0.05, **p<0.0005):  All items:  SP = 9.88 (3.44)  Vignette: 10.04 (3.37)  Obligatory items**:  SP = 7.04 (1.54)  Vignette: 5.24 (1.80)  Intermediate*:  SP = 1.96 (1.24)  Vignette: 2.76 (1.36)  Superfluous*:  SP = 0.88 (1.74)  Vignette: 2.04 (2.16) |
| 17. Rethans 1994 Netherlands | Approached: 39 GPs  Excluded: Four (one relocated, three agreed to pilot phase only)  Enrolled:35  Analysed: Frequencies/mean scores 35 GPs. Correlations 18 GPS who returned data for all four SP conditions.  Percentage analysed: 90  Mean age (yrs): NR  Percentage male: NR | Item by item and summary scores  Frequencies, mean and IQR of sensitivity for several categories of behaviour. Pearson’s product moment correlation across the four SP conditions for six categories, for SP report and medical record entries: History taking, physical examination, laboratory examination, guidance and advice, medication and therapy, follow-up. | ‘Content Score’: Mean (IQR):  Overall (all categories and all conditions): 0.32 (0.27 to 0.37). (*i.e.*, only 32% of all actions actually undertaken were recorded in medical records)  Correlation coefficient:  Overall (all categories and all conditions) = 0.54 (p<0.05)  History taking = 0.17  Phys examination = 0.45  Lab examination = 0.75 (p<0.01)  Guidance and advice = 0.50 (p<0.05)  Medication and therapy = 0.43  Follow-up = -0.04 |
| 18. Peabody 2000 USA | Approached: 101 physicians  Not consented: 3  Enrolled: Randomly selected: 20 (10 physicians from each medical centre)  Analysed: 20 physicians and 160 summary scores (8 cases for each physician)  Percentage analysed: 100  Mean age (yrs): NR  Percentage male: NR | Summary scores.  Scores for the three methods were compared using a four-way (three-way nested, one-way crossed) ANOVA model. Factors were design effects and random effects. A site-method interaction term was included. Sub-analyses assessed case effects; site and provider effects; domains; cuing and detection effects. | Summary scores (% correct responses (SD)):  All conditions:  SP = 76.2 (7.2)  Vignette = 71.0 (5.4)  Medical records = 65.6 (8.7)  Four-way ANOVA p-value:  p<0.001  Summary scores also presented for each of 4 conditions: SP scores were consistently higher than vignettes. Vignettes were consistently higher than charts, except for CAD.  Four-way ANOVA p-value:  LBP: p<0.001  COPD: p = 0.002  DM: p = 0.001  CAD: p = 0.05  Interaction effect was not significant (p = 0.14) |
| 19. O’Boyle 2001 USA | Approached: 474  Enrolled: 124  Analysed: 120 Nurses Percentage analyzed: 97  Mean age (yrs): NR  Percentage male: NR | Summary scores.  Pearson correlation coefficients. Structural equation modelling. No adjustment for clustering. The differences in self-reported adherence rates among the hospitals were significant (p<0.001). The dataset available for analysis was ‘complete’. | Mean % (SD) adherence score:  Observed: 69.97 (21.88)  Self-reported: 81.7 (15.68)  Pearson correlation coefficient: r = 0.39  SEM coefficient = 0.201 (p<0.05) |
